# THE IMPACT OF EDUCATIONAL LEVEL ON FOOD SECURITY AMONG OLDER ADULTS AMID THE COVID-19 PANDEMIC

**DOI:** 10.1093/geroni/igae098.2340

**Published:** 2024-12-31

**Authors:** Lillie Monroe-Lord, Azam Ardekani, Phronie Jackson, Cassidy Weitman

**Affiliations:** University of the District of Columbia, Washington, District of Columbia, United States; University of the District of Columbia, Washington, District of Columbia, United States; University of the District of Columbia, Washington, District of Columbia, United States; University of the District of Columbia, Washington, District of Columbia, United States

## Abstract

The COVID-19 pandemic has dramatically altered global health and economic landscapes, significantly threatening food security worldwide. This study investigates the pandemic’s impact on perceived food security among older adults, with a particular focus on the role of educational attainment. A total of 4,961 participants aged 65 and above were surveyed between August 9 and September 15, 2020, utilizing the USDA’s Six-Item Short Form via Qualtrics to measure food security. The survey, conducted retrospectively twice, assessed higher scores as indicative of increased food insecurity among varying educational backgrounds: less than a high school education, some college education, and a college or graduate degree. Findings indicated older adults with less than a high school diploma were most affected, experiencing a mean percentage increase in food insecurity of 7.7% (p = 0.007). Respondents with some college education saw a 7.03% increase, which was not statistically significant (p > 0.05). Conversely, older adults with a college or graduate degree reported a smaller, yet statistically significant, increase in food insecurity (4.06%, p = 0.041). These results highlight a correlation between educational level and food security, with lower levels of education being associated with a greater risk of food insecurity during the COVID-19 pandemic. This study underscores the need for enhanced support systems to protect the most vulnerable populations, especially the less educated older adults, from the adverse effects of global health emergencies on food security.

